# Integrin-Linked Kinase Is a Functional Mn^2+^-Dependent Protein Kinase that Regulates Glycogen Synthase Kinase-3β (GSK-3β) Phosphorylation

**DOI:** 10.1371/journal.pone.0012356

**Published:** 2010-08-23

**Authors:** Mykola Maydan, Paul C. McDonald, Jasbinder Sanghera, Jun Yan, Charalampos Rallis, Sheena Pinchin, Gregory E. Hannigan, Leonard J. Foster, David Ish-Horowicz, Michael P. Walsh, Shoukat Dedhar

**Affiliations:** 1 Department of Integrative Oncology, BC Cancer Research Centre, Vancouver, British Columbia, Canada; 2 SignalChem Inc., Richmond, British Columbia, Canada; 3 Developmental Genetics Laboratory, London Research Institute, London, United Kingdom; 4 Department of Genetics, Evolution & Environment and UCL Cancer Institute, University College London, London, United Kingdom; 5 Centre for Cancer Research, Monash Institute of Medical Research, Melbourne, Victoria, Australia; 6 Department of Biochemistry and Molecular Biology, University of British Columbia, Vancouver, British Columbia, Canada; 7 Department of Biochemistry and Molecular Biology, University of Calgary, Calgary, Alberta, Canada; Universität Heidelberg, Germany

## Abstract

**Background:**

Integrin-linked kinase (ILK) is a highly evolutionarily conserved, multi-domain signaling protein that localizes to focal adhesions, myofilaments and centrosomes where it forms distinct multi-protein complexes to regulate cell adhesion, cell contraction, actin cytoskeletal organization and mitotic spindle assembly. Numerous studies have demonstrated that ILK can regulate the phosphorylation of various protein and peptide substrates *in vitro*, as well as the phosphorylation of potential substrates and various signaling pathways in cultured cell systems. Nevertheless, the ability of ILK to function as a protein kinase has been questioned because of its atypical kinase domain.

**Methodology/Principal Findings:**

Here, we have expressed full-length recombinant ILK, purified it to >94% homogeneity, and characterized its kinase activity. Recombinant ILK readily phosphorylates glycogen synthase kinase-3 (GSK-3) peptide and the 20-kDa regulatory light chains of myosin (LC_20_). Phosphorylation kinetics are similar to those of other active kinases, and mutation of the ATP-binding lysine (K220 within subdomain 2) causes marked reduction in enzymatic activity. We show that ILK is a Mn-dependent kinase (the K_m_ for MnATP is ∼150-fold less than that for MgATP).

**Conclusions/Significance:**

Taken together, our data demonstrate that ILK is a *bona fide* protein kinase with enzyme kinetic properties similar to other active protein kinases.

## Introduction

Integrin-linked kinase (ILK) was discovered as an interactor of integrin β1 and β3 cytoplasmic domains [1], which localizes to focal adhesion plaques and to centrosomes [2], [3], and is composed of three distinct domains: an *N*-terminal ankyrin-repeat domain, a pleckstrin homology (PH)-like domain and a kinase catalytic domain [2]. The domains are highly evolutionarily conserved, and genetic studies in model organisms such as *C.elegans*, *Drosophila*, *Xenopus laevis*, zebrafish, and mice have demonstrated an essential role of ILK in embryonic development and diverse physiological functions, and have confirmed tight functional linkages of ILK with integrins [2], [4]. Inhibition of ILK kinase activity has significant effects on cell survival and proliferation in cancer cells relative to most normal cells [5], suggesting that, although ILK kinase activity may be silenced in most normal cells, it becomes activated in cancer cells where it is required for cell survival and proliferation.

The kinase domain of ILK is atypical in that it lacks the highly conserved Asp-Phe-Gly (DFG) and His-Arg-Asp (HRD) motifs, but possesses the invariant Lys residue involved in ATP binding, as well as the invariant Ala-Pro-Glu (APE) motif. This has resulted in the classification of ILK as a pseudokinase [2], [6]. Recent structure-function analyses of other “pseudokinases”, such as CASK and haspin [7], [8], both of which lack the DFG motif (haspin also lacks the APE motif), have clearly demonstrated that these kinases are catalytically functional. ErbB3, a member of the epidermal growth factor receptor family and long thought to be inactive as a kinase, has now been shown to be catalytically active [9], [10]. These studies have identified unusual mechanisms of catalysis for these enzymes, thereby challenging a simplistic categorization of kinases based on amino acid sequence alone.

Kinase assays using either recombinant wild-type or mutant ILKs, or ILK purified from tissues or immunoprecipitated from mammalian cellular lysates have shown that ILK can regulate the phosphorylation of several protein substrates, e.g., integrin β1 cytoplasmic domain [1], the ILK-binding protein, β-parvin [11], Akt/PKB [5], [12]–[25], GSK-3β [24], [26]–[35], the 20-kDa regulatory light chains of myosin, LC_20_ [36]–[40], the myosin targeting subunit of myosin light chain phosphatase, MYPT1 [38], [39], protein phosphatase inhibitors, PHI-1, KEPI and CPI-17 [36], [37], [41], [42], and α-NAC [20]. Indeed, ILK was discovered completely independently as a calcium-independent myosin light chain protein kinase [40], as well as a kinase capable of directly phosphorylating MYPT1 [38], [39] and the CPI-17 family of protein phosphatase inhibitors [42]. In addition, ILK-null mutants in zebrafish are rescued by wild-type, kinase-active ILK, but not by ILK containing mutations within the kinase domain [13], and kinase-deficient mutants of ILK have been identified in the myocardium of patients with dilated cardiomyopathy [43].

However, other studies have shown that kinase domain mutations can support normal embryonic development and rescue genetic defects *in vivo* [44], [45], challenging the requirement of kinase activity for ILK function. The *C*-terminal catalytic and the *N*-terminal ankyrin domains of ILK interact directly with several proteins, suggesting that ILK fulfils an adaptor/scaffolding function in addition to a kinase function. These complex protein-protein interactions [2], [4], [46] are also likely to regulate the kinase activity of ILK, as indicated by the recent structure determination of a complex between the ILK kinase domain and the *C*-terminal calponin homology (CH2) domain of α-parvin [47]. Thus, controversy persists as to whether ILK can function as a protein kinase [45], especially since the structure of the full-length, uncomplexed protein has yet to be solved, and rigorous characterization of the kinase activity of purified ILK is lacking.

To address these issues, we have expressed and purified full-length wildtype and mutant ILKs in the baculovirus/insect cell system and characterized their kinase activities. We show that ILK is indeed a protein kinase capable of phosphorylating protein and peptide substrates with comparable kinetics to those of other protein kinases. We find that ILK has a strong preference for the Mn^2+^ divalent cation. We also show that the ATP-coordinating lysine residue, K220, is required for full activation of ILK *in vitro*. ILK kinase activity is inhibited by interaction with its binding partner, α-parvin. Together, these results argue that ILK is not a pseudokinase, but an authentic protein kinase.

## Results

### Recombinant ILK phosphorylates GSK-3 crosstide and the 20 kDa regulatory light chain of myosin, LC_20_

**Figure 1:**
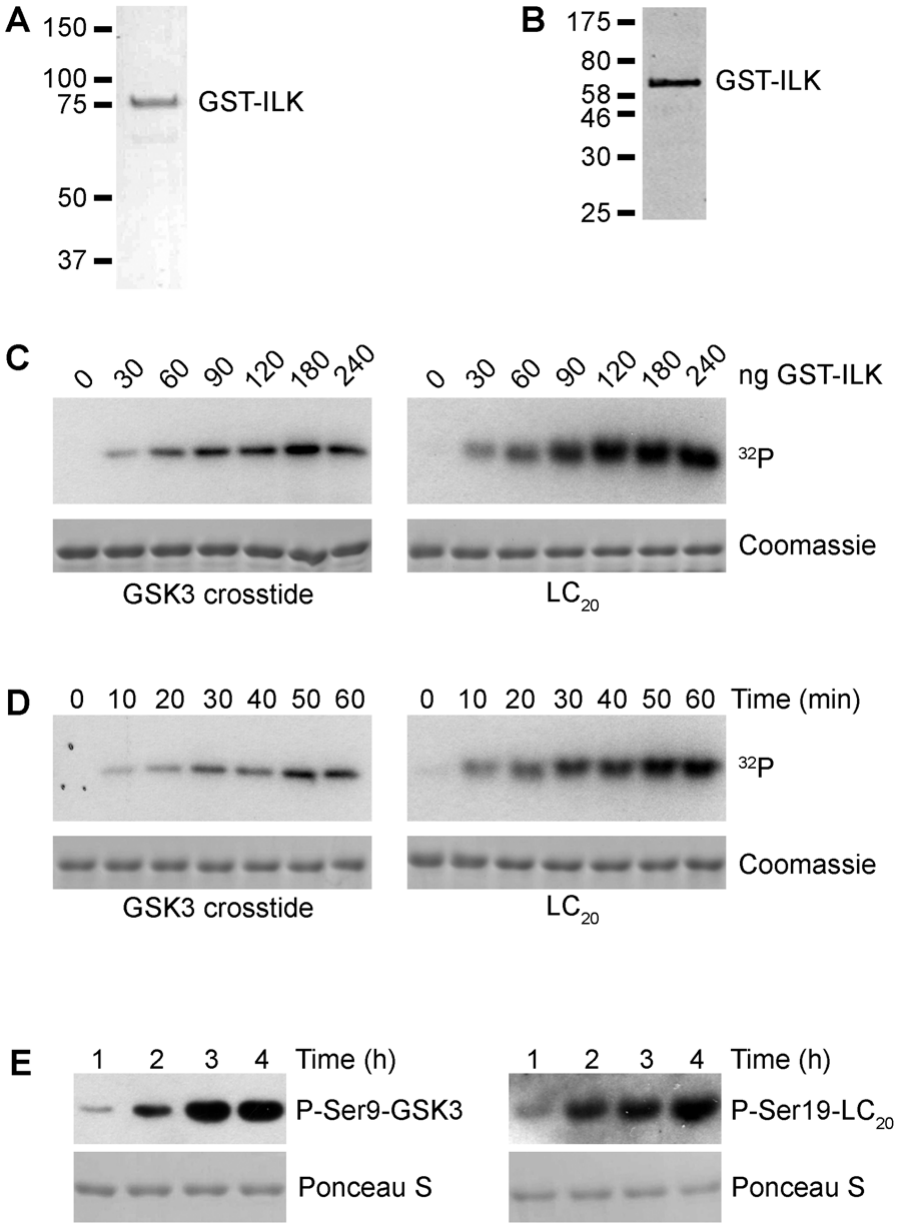
Purified GST-ILK is an active serine kinase. (A) Coomassie blue-stained polyacrylamide gel showing a single protein band at approximately 78 kDa, the predicted molecular weight of the GST-ILK fusion protein, prepared as described in *Materials and Methods*. (B) Western blot analysis using antibodies specific for ILK confirm the identity of the 78 kDa band. (C) Analysis of the concentration dependence of the protein kinase activity of GST-ILK using GSK-3 crosstide and LC_20_ as substrates. Experiments were carried out using standard kinase reaction conditions (described in *Materials and Methods*) in the presence of 10 mM MgCl_2_. Representative autoradiographs (upper panels) show the amount of ^32^P-phosphate incorporation with increasing concentrations of GST-ILK. Coomassie stains of SDS-gels (lower panels) depict equal loading of substrate into the kinase reaction. (D) Time course of the protein kinase activity of GST-ILK. Reactions were performed as described in C using GSK3 crosstide and LC_20_ as substrates. The amount of ILK was held constant at 30 ng. (E) Phosphorylation of Ser9 on GSK3 crosstide and Ser19 on LC_20_. Each substrate was incubated with 150 ng of GST-ILK in 25 µl of kinase reaction buffer (see *Materials and Methods*) for the indicated times in the presence of 500 µM cold ATP. Western blot analysis was carried out using phospho-specific antibodies as described in *Materials and Methods*. Ponceau S stains of membranes are provided as loading controls.

A GST-ILK construct was cloned into baculovirus and GST-ILK fusion protein was expressed in insect cells and purified as described in *Materials and Methods*. As shown in Fig. 1A, separation of the GST-ILK preparation by SDS-PAGE and staining of the gel with Coomassie blue showed the presence of a full-length protein with the expected MW of approximately 78 kDa and purity of >94%. Western blot analyses with anti-ILK (Fig. 1B) confirmed the identity of the band as GST-ILK. Although very few other proteins were detected on the stained gel (Fig. 1A), we analyzed the total protein complement of the ILK preparation by mass spectrometry as described in *Materials and Methods* to ensure that there were no other protein kinases present. As shown in Table S1, although several insect proteins were minor contaminants of the preparation, no additional protein kinases were detectable, ensuring that any protein kinase activity detected is due to ILK. The presence of human ILK protein was also confirmed by mass spectrometry with greater than 94% sequence coverage, as shown in Fig. S1.

To analyze protein kinase activity, we utilized two well characterized substrates of ILK, GST-GSK-3 α/β crosstide and LC_20_. The GSK-3 crosstide is a commercially available peptide, previously identified as a substrate of ILK [2], [24], [29]. The phosphorylation of glycogen synthase kinase (GSK)-3β itself on Ser-9 has been shown to be regulated by ILK in several cellular systems [24], [27], [29], [33]. Phosphorylation inactivates GSK-3β, resulting in the stimulation of several downstream signaling pathways that regulate cell proliferation and cell survival [33], [35], [48]. LC_20_ has previously been shown to be directly phosphorylated by ILK purified from smooth muscle tissue [40]. Experiments were performed using standard kinase reaction conditions, including the use of Mg^2+^ and ATP, as outlined in *Materials and Methods*. As shown in Fig. 1C, ILK readily phosphorylates GSK-3 crosstide and LC_20_ in a manner dependent on the concentration of ILK. The phosphorylation of LC_20_ and GSK-3 crosstide by ILK also occurred in a time-dependent fashion (Fig. 1D).

We next determined whether recombinant ILK phosphorylates GSK-3 crosstide and LC_20_ at the previously identified serine residues [27], [29], [31], [33] by carrying out kinase assays with non-radioactive ATP. Phosphorylation of the substrates was detected by Western blot analysis using phospho-specific antibodies. As shown in Fig. 1E, ILK phosphorylates GSK-3 crosstide at a serine residue corresponding to serine 9 in GSK-3β and serine 21 in GSK-3α. A large number of studies utilizing ILK dominant-negative mutants, siRNA and small molecule inhibitors have reported that ILK can regulate GSK-3 phosphorylation on Ser9/21 [reviewed in 2]. Other studies have reported direct phosphorylation of GSK-3 by recombinant GST-ILK [24], [33]. LC_20_ was also identified as a substrate of ILK, and shown to be phosphorylated at Thr-18 and Ser-19 [40]. As shown in Fig. 1E, recombinant ILK directly phosphorylates LC_20_ on serine 19. These data demonstrate that ILK directly phosphorylates physiologically relevant substrates *in vitro*.

### ILK autophosphorylation

**Figure 2:**
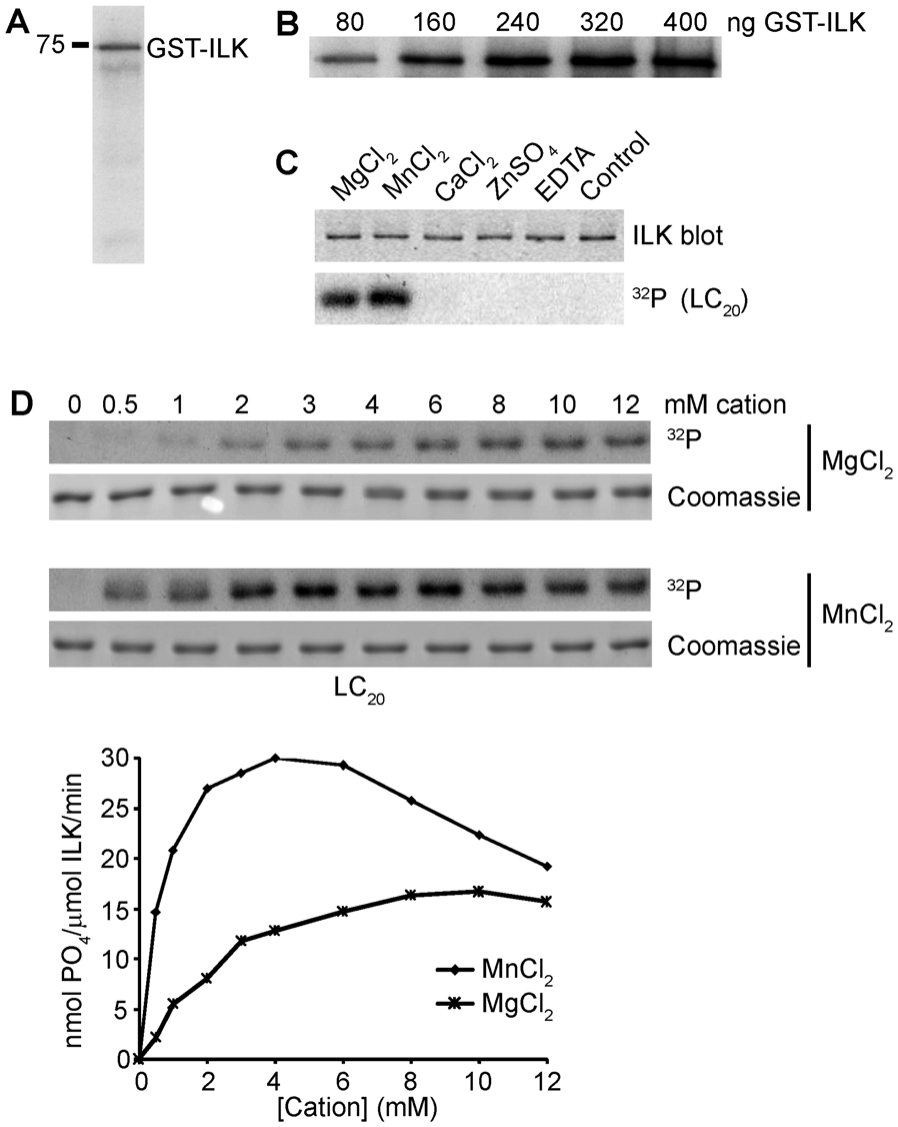
ILK autophosphorylation and divalent cation requirements for ILK activity. (A) Autoradiograph showing autophosphorylation of GST-ILK. Reactions were carried out in the absence of exogenous substrate for 60 min using 80 ng of ILK in the presence of 5 mM MnCl_2_. (B) Concentration-dependent increase in autophosphorylation of ILK. An autoradiograph is shown to demonstrate that the amount of autophosphorylation increases with increasing amounts of ILK. Reactions were carried out as described in A using the indicated amounts of ILK. (C) Autoradiograph demonstrating ILK kinase activity in the presence of a variety of divalent cations. Reactions were carried out for 30 min using 30 ng of ILK. LC_20_ was used as the substrate. A Western blot for ILK demonstrates the presence of equal amounts of enzyme in each reaction. (D) The effects of Mg^2+^ and Mn^2+^ on the velocity of ILK activity. Top, autoradiographic images and Coomassie stains demonstrating increased kinase activity of ILK in MnCl_2_. Reactions were carried out for 30 min using 30 ng of ILK and the indicated concentrations of each cation. LC_20_ was used as the substrate. Bottom, quantification of the radiographic data. A plot of reaction velocity against increasing concentrations of the indicated cations is shown.

Most protein kinases are capable of autophosphorylation [7], and autophosphorylation activity is frequently employed as a test for kinase activity [49]. To determine whether ILK can undergo autophosphorylation, we carried out kinase reactions in the absence of exogenous substrates. As shown in Fig. 2A and 2B, ILK is readily autophosphorylated in a concentration-dependent manner.

### Enzyme kinetics

The analysis of the kinase activity of another apparent “pseudokinase”, CASK [7], demonstrated unusual divalent cation requirements for catalytic activity. Indeed, CASK activity is inhibited by divalent cations and is constitutively active in the absence of cations. Coordination of the β and γ phosphates of ATP by Mg^2+^ ions catalyzes phosphotransfer reactions by most protein kinases [50], [51]. We therefore characterized the divalent cation requirement of ILK kinase activity. Kinase activity was readily detected in the presence of MgCl_2_ and MnCl_2_, but not in the presence of CaCl_2_, ZnSO_4_ or EDTA (Fig. 2C), demonstrating a requirement for Mg^2+^ and Mn^2+^ cations.

We compared the differential effects of Mg^2+^ and Mn^2+^ ions on the kinase activity of the recombinant ILK protein. As shown in Fig. 2D, the reaction velocity was significantly higher in the presence of MnCl_2_ than MgCl_2_. ILK activity for LC_20_ was markedly enhanced by low concentrations of Mn^2+^, peaking at approximately 4–5 mM MnCl_2_, and then declined gradually at higher concentrations.

**Figure 3:**
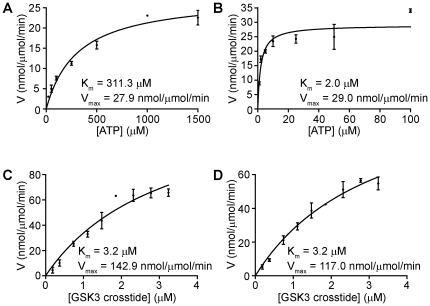
Michaelis-Menten (MM) kinetics of GST-ILK. (A) MM plot of reaction velocity against increasing concentrations of ATP in the presence of 10 mM MgCl_2_. Reactions were carried out for 30 min using 30 ng of ILK and 1 µg of GSK-3 crosstide as the substrate. (B) MM plot of reaction velocity against increasing concentrations of ATP in the presence of 5 mM MnCl_2_. Reactions were carried out as described in A. (C) MM plot of reaction velocity against increasing concentrations of GSK-3 crosstide in the presence of 10 mM MgCl_2_. Reactions were carried out for 30 min using 30 ng of ILK. The ATP concentration was 500 µM. (D) MM plot of reaction velocity against increasing concentrations of GSK3 crosstide in the presence of 5 mM MnCl_2_. Reactions were carried out for 30 min using 30 ng of ILK. The ATP concentration was 500 µM. For all data points, values are reported as means ± SEM, n = 3. K_m_ and V_max_ values were calculated as described in *Materials and Methods*.

We used Michaelis-Menten kinetics to determine the K_m_, V_max_, and V_max_∶K_m_ values for ATP and GSK-3 crosstide. As shown in Fig. 3A and 3B, and Table 1, the K_m_ for ATP is `150-fold lower in the presence of MnCl_2_ (2.0 µM) than MgCl_2_ (311 µM). Similarly, the V_max_∶K_m_ ratio is significantly higher in MnCl_2_ than in MgCl_2_ (Table 1), demonstrating a two order of magnitude increase in enzymatic efficiency in the presence of manganese. We also determined the K_m_ for GSK-3 crosstide in the presence of MgCl_2_ or MnCl_2_ (Figs. 3C and 3D). The calculated K_m_ values (∼3.2 µM) are entirely within the range of K_m_ values of other active protein kinases [7], and compare well with the reported K_m_^ATP^ values of CASK [7] and haspin [8], both of which were previously proposed to be pseudokinases. The calculated V_max_ and V_max_∶K_m_ values (Table 1) are also comparable [7], [8]. These data indicate that ATP coordination is sensitive to divalent cations, being much more efficient in MnCl_2_ compared to MgCl_2_. However, in saturation conditions of ATP, the K_m_ for substrate (GSK-3 crosstide) is similar in the presence of either divalent cation. These data demonstrate that ILK is an active protein kinase, whose overall kinetic properties compare favorably with those of other active protein kinases.

**Table 1:** Michaelis-Menten enzyme kinetics for GST-ILK.

	K_m_ (µM)	V_max_ (nmol/µmol/min)	V_max_∶K_m_ (×10^−5^)
**Mg^2+^/ATP**	311.3	27.9	8.9
**Mn^2+^/ATP**	2.0	29.0	1450
**GSK3 (Mg^2+^)**	3.2	142.9	4465
**GSK3 (Mn^2+^)**	3.2	117.0	3656

Substrate and enzyme velocity data were fit to a Michaelis-Menten model and V_max_ and K_m_ were calculated using the Michaelis-Menten model features in GraphPad Prism 5.00 for Windows (GraphPad Software, San Diego, CA). To calculate the ratio of V_max_∶K_m_, the values for Vmax were transformed from nmol/µmol/min to µmol/µmol/min.

### α-Parvin inhibits ILK kinase activity

The recently reported crystal structure of the kinase domain of ILK in complex with α-parvin shows that this complex, although capable of binding ATP, is catalytically inactive, and that the kinase domain of ILK can function as a scaffold for protein-protein interactions [47]. However, these studies used suboptimal assay conditions, in particular ILK was incubated with protein or peptide substrates, Mg^2+^ and unlabeled ATP prior to starting the reaction with radiolabeled ATP. In this case, the protein substrate will be phosphorylated by cold ATP so that addition of [γ-^32^P]ATP will fail to incorporate [^32^P]phosphate. Also, no quantification of the data was presented.

**Figure 4:**
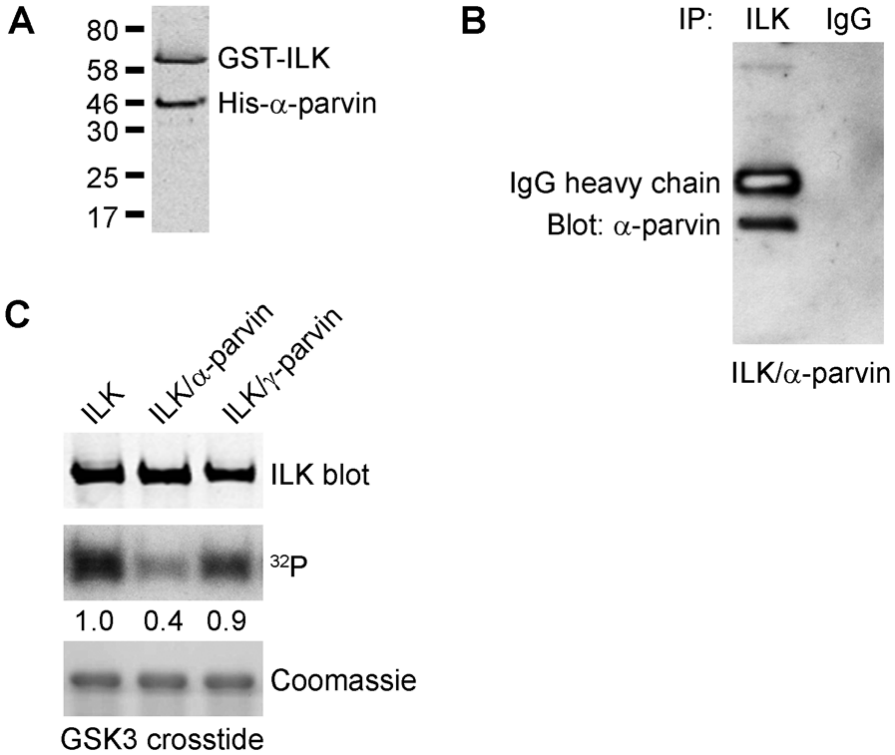
Binding of α-parvin inhibits ILK kinase activity. (A) Western blot showing co-expression of GST-ILK and His-α-parvin using antibodies to ILK and α-parvin. (B) Co-immunoprecipitation of ILK and α-parvin. The complex of ILK and α-parvin was immunoprecipitated using ILK and blotted using antibodies to α-parvin. Non-immune IgG served as a control. (C) Autoradiograph showing kinase activity of ILK alone, ILK complexed with α-parvin (ILK/α-parvin) or ILK complexed with γ-parvin (ILK/γ-parvin). Reactions were carried out for 30 min using 30 ng of each enzyme preparation and GSK-3 crosstide as the substrate. Western blot analysis for ILK and Coomassie stains of SDS-gels are provided as loading controls for equal amounts of ILK and GSK-3 crosstide substrate, respectively.

It is conceivable that the “inactive” conformation and the reduced ILK activity detected by Fukuda et al [47] is due to α-parvin exerting a constraint on the kinase domain and locking it into an inactive conformation. We therefore investigated whether the interaction of α-parvin with ILK *in vitro* can directly modulate ILK kinase activity by purifying the ILK/α-parvin complex from cells co-expressing full-length ILK and α-parvin via the baculovirus system. As shown in Fig. 4A, ILK was co-expressed with α-parvin and the two proteins were demonstrated to exist as a complex as shown by co-immunoprecipitation (Fig. 4B). Interestingly, the ILK/α-parvin complex is significantly less active (Fig. 4C) than ILK alone, while the ILK-γ-parvin complex exhibited a level of activity approaching that of wildtype ILK, suggesting that distinct parvins could modulate ILK kinase activity under physiological and pathological conditions *in vivo* [2], [4].

### The ATP-binding Lysine 220 is required for ILK kinase activity

Although ILK lacks the Mg^2+^-ATP coordinating motif, DFG, as well as the HRD motif within the activation loop involved in phosphotransfer, ILK possesses the invariant ATP-coordinating lysine residue within subdomain 2 [1], K220, as well as the invariant APE motif. Mutation of K220 to alanine or methionine has shown that K220 is essential for ILK function *in vitro* and *in vivo*. Specifically, K220 of ILK has been shown to be essential for kidney development and function [45], in enhancing adhesion and focal adhesions during bacterial colonization of epithelial cells [52], and in cardiac function [13], [43]. Although the effect of the “knock-in” K220M mutation of ILK on renal development and function was attributed to an adaptor role of ILK in impaired parvin interaction [45], it is equally likely that it is due to impaired kinase activity. Indeed, the crystal structure of the ILK kinase domain in complex with α-parvin indicates that the K220 ATP-binding motif is well separated from the α-parvin-contacting residues, M402 and K403 in the G helix, and parvin does not have a direct effect on ATP binding [47], making it unlikely that the major developmental effect of mutating K220 is solely through α-parvin binding.

**Figure 5:**
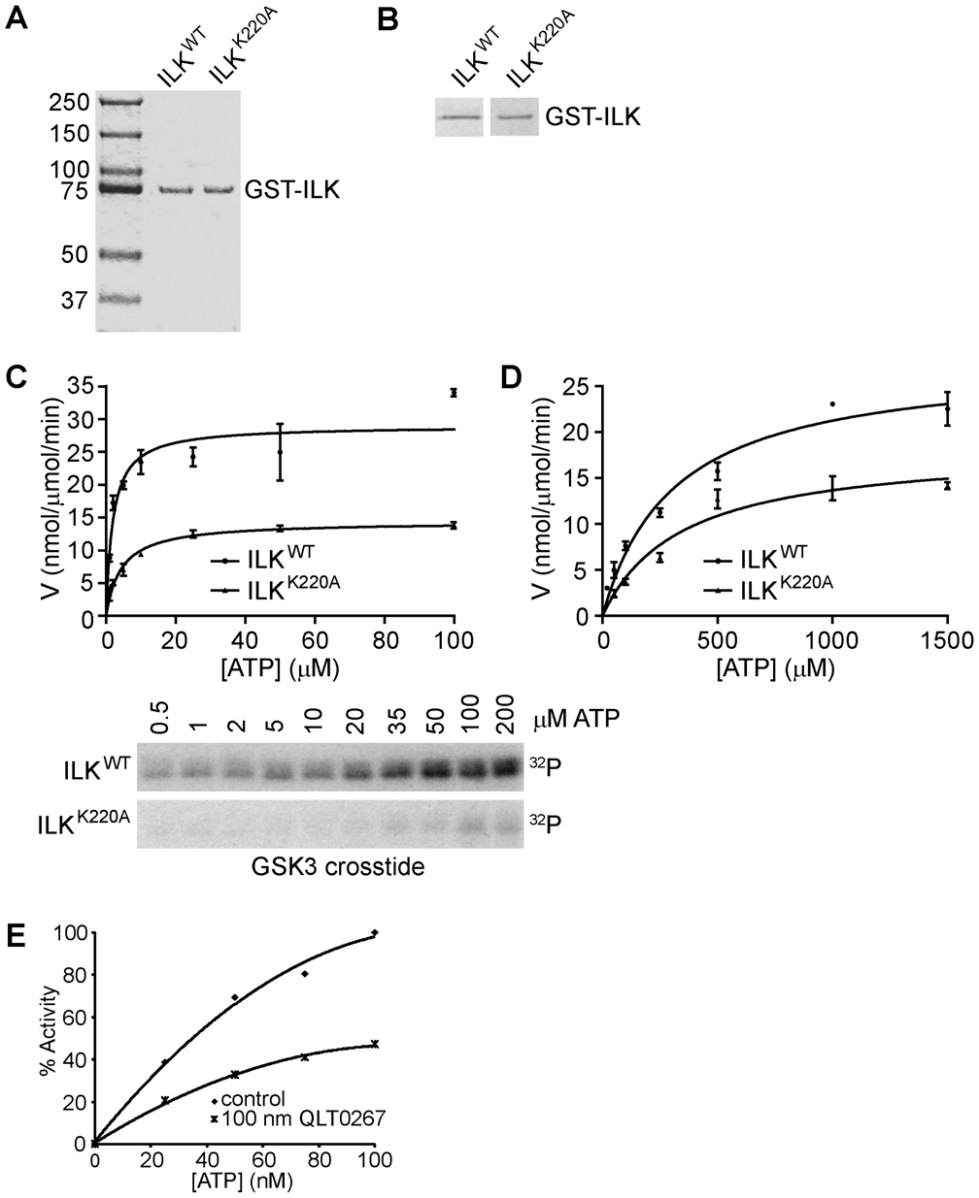
ILK activity is reduced by mutation of K220 and by a specific pharmacological inhibitor, QLT0267, and is required for Ser9 phosphorylation of GSK-3β in vivo. (A) Coomassie-stained SDS-gel showing equal amounts of wild-type GST-ILK (ILK^WT^) and mutant GST-ILK (ILK^K220A^) prepared as described in *Materials and Methods*. (B) Western blot analysis using antibodies specific for ILK to confirm the identities of ILK^WT^ and ILK^K220A^. Western blot analysis confirmed the presence of equal amounts of protein for both constructs. (C) Top, plot of reaction velocity with increasing ATP concentrations for ILK^WT^ and ILK^K220A^ in 5 mM MnCl_2_. Bottom, autoradiographic images demonstrating ILK kinase activity for ILK^WT^ and ILK^K220A^ with increasing ATP concentrations. Reactions were carried out for 30 min using 30 ng ILK and GSK3 crosstide as the substrate. (D) Plot of reaction velocity with increasing ATP concentrations for ILK^WT^ and ILK^K220A^ in 10 mM MgCl_2_. Reaction conditions were as described in C. (E) Kinase activity of GST-ILK in the presence of 100 nM QLT0267, a specific small molecule inhibitor of ILK. Reactions were carried out for 30 min using 30 ng ILK with GSK3 as the substrate.

Therefore, we investigated whether Lys-220 is indeed required for ILK catalytic function by analyzing recombinant protein in which this residue is mutated to a non-coordinating alanine. We prepared K220A ILK (see *Experimental procedures*) and compared its kinase activity with that of wild-type ILK under the optimal conditions established in this study. As shown in Fig. 5A and 5B, wild-type and K220A ILK, identified with anti-ILK, are expressed at comparable levels (Fig. 5B). The K220A mutation was confirmed by DNA sequencing (see *Materials and Methods*). As shown in Fig. 5C, the kinase activity of the K220A ILK mutant in the presence of Mn^2+^ is significantly reduced over a range of ATP concentrations relative to that of wild-type ILK, demonstrating that K220 is indeed required for maximal catalytic activity of ILK. Similar results were obtained when the reactions were carried out in the presence of Mg^2+^ (Fig. 5D).

Small molecule inhibitors of ILK kinase activity have been developed and characterized *in vitro* and *in vivo* [5], [53], [54]. Because of the robust catalytic activity of ILK characterized here, we wanted to confirm the inhibitory activity of QLT-0267 [5] under optimal kinase reaction conditions. As shown in Fig. 5E, QLT-0267 inhibits ILK activity in an ATP-competitive manner. In addition, QLT-0267 was found to be the most potent inhibitor of ILK activity as compared to several other kinase inhibitors (Fig. S2).

## Discussion

The data presented in this paper establish that ILK is an active serine/threonine protein kinase despite having an atypical kinase domain. We have conducted a detailed kinetic analysis of the kinase activity of wildtype ILK and of the K220A mutation that is unable to co-ordinate ATP, and determined that ILK is significantly more active in the presence of Mn^2+^ than Mg^2+^ ions. These findings have implications for the regulation of ILK kinase activity *in vivo*.

Recent structure-function analyses of other “pseudokinases”, such as CASK [7] and haspin [8], which have atypical kinase domains similar to ILK, have clearly demonstrated that atypical kinase domains can be functional and are capable of catalysis. Novel mechanisms for kinase activity have been identified through these structure-function analyses [8], [55]. The structure of the ILK kinase domain has recently been solved, albeit as a complex with α-parvin, resulting in an inactive conformation and demonstrating that this domain of ILK can form a bi-lobed kinase-like conformation and can bind ATP, but can also function as a protein scaffold.

Here, we have established that purified recombinant full-length ILK is catalytically active, capable of phosphorylating several substrates such as GSK-3 and LC_20_, both of which have previously been demonstrated to be physiological substrates of ILK (reviewed in 2). We have also established that the K_m_ values for ATP and peptide or protein substrates of ILK are within the range of other protein kinases [7], [8], and that the K_m_ values of ILK for MnATP are much lower, and the V_max_∶K_m_ much higher than for MgATP, and comparable to those of CASK and haspin [7], [8]. The differential effects of manganese and magnesium on ILK kinase activity are interesting and are very similar to those described recently for the kinase activity of leucine-rich repeat kinase 2 (LRRK2) [56]. Together, these results suggest that ILK is a more efficient kinase in the presence of manganese.

We have also established the important role of Lys-220 for the enzymatic activity of ILK. This invariant lysine residue in subdomain 2 coordinates ATP, and mutation to alanine results in significant inhibition of catalytic activity. It is surprising that the mutation of K220 did not completely abrogate activity, but instead resulted in a substantially less active kinase. The reasons for this are unclear at present, although a similar reduction in activity was noted when the analogous lysine residue was mutated in haspin [8]. Our finding that mutation of K220 results in a significantly less active kinase is important with regard to the interpretation of a recent genetic study in which knock-in of the K220M mutant resulted in a dramatic kidney development defect [45] likely due, at least in part, to defects in renal branching morphogenesis [57]. The interpretation of the authors was that this mutant fails to bind α-parvin, resulting in defective function, and it was suggested that K220 represents a binding site for α-parvin. However, as we have shown that this mutation also results in a significant reduction in ILK activity, it is entirely possible that the defects in the kidney are due to a requirement for kinase activity, especially since ILK activity and GSK-3 phosphorylation have been shown to be required for branching morphogenesis of kidney tubular epithelial cells [57].

Also, it is unclear how K220 would be involved in α-parvin binding since the ILK kinase domain-parvin structure shows that the parvin-contacting residues, M402 and K403 in the G helix, are very distant from the K220 ATP-coordinating amino acid in the nucleotide-binding cleft [47]. This result is consistent with our data showing loss of catalytic activity of the K220A mutant towards exogenous substrates. We have also demonstrated here that α-parvin binding to ILK results in significant inhibition of kinase activity, consistent with the inactive conformation of the parvin-bound kinase domain described by Fukuda et al [47].

Fukuda et al [47] concluded that ILK does not exhibit kinase activity based on the inactive conformation of the ILK kinase domain in complex with the CH2 domain of α-parvin, and their inability to detect phosphorylation of putative substrates (myelin basic protein, integrin β1 and β3 *C*-termini fused to maltose-binding protein, His-tagged α-parvin CH2 domain, and GST-Akt) by bacterially-expressed, full-length ILK in complex with the LIM1-2 domain of PINCH. Our results, on the other hand, clearly demonstrate that the conclusion that ILK is a pseudokinase [6], [58] is not valid. Here, we have provided unequivocal data that demonstrate that purified recombinant ILK is active as a serine/threonine protein kinase *in vitro*.

The regulation of the kinase activity of ILK *in vivo* is likely to be dynamic and complex, depending on multiple factors such as protein-protein interactions and subcellular localization, and the availability of divalent cations, in particular Mn^2+^, as well as phosphoinositides. Although the cellular concentrations of manganese are much lower than magnesium, the concentrations of divalent cations may vary significantly in different subcellular niches, likely making ILK more or less active in different protein complexes. Furthermore, our results, when put into context with other reports, clearly point to tissue-specificity of the kinase *versus* adapter roles of ILK *in vivo*, and these functions are likely not mutually exclusive. For example, the binding of α-parvin to the ILK catalytic domain results in the inhibition of the kinase activity, suggesting that dynamic protein-protein interactions within cells can regulate ILK kinase activity. Tissue-specific knock out of ILK, as well as tissue specific modulation of ILK using kinase-deficient dominant-negative constructs, have clearly shown a requirement of kinase function *in vivo*, for example for neuronal polarity and dendrite and axon growth [26], [29], [59] and cardiac function [13], [43].

Future studies aimed at solving the crystal structure of full-length, uncomplexed ILK, as well as various ILK complexes will be necessary to understand the catalytic mechanism and complex regulation of this atypical protein kinase. To paraphrase a recent review on ILK activity [58], the pseudokinase is dead, long live the kinase!

## Materials and Methods

### Cloning and purification of GST-ILK, GST-ILK^K220A^, and His-α-parvin constructs

DNA primers for human *ILK* (NM_004517) and *α-parvin* (NM_018222) were synthesized, the cDNAs cloned by PCR and the sequences of the products verified by DNA sequencing. The sequences of the primers and GST fusion proteins are provided in *Methods S1*. The verified ILK gene product was sub-cloned into the *pAcG2T* plasmid (BD PharMingen) for expression in insect cells. The cloned α-parvin gene product was similarly sub-cloned into the *pAcSPHis* plasmid (BD PharMingen). The human *ILK^K220A^* cDNA was generated by site-directed mutagenesis as described previously [23], and was sub-cloned into baculovirus expression plasmid *pAcSPG4T* (BD PharMingen). Highly purified plasmid DNAs containing *ILK*, *ILK^K220A^*, or α-parvin were then prepared using a miniprep DNA purification kit (QIAGEN).

### Transfection and amplification of recombinant baculovirus in Sf9 cells

Purified *pAcG2T-ILK, pAcSPG4T-ILK^K220A^*, and *pAcSPHis-α-parvin* plasmids were transfected separately with linear *AcNPV* DNA (BD PharMingen) into *Spodoptera frugiperda* (Sf9) cells (Invitrogen) to produce recombinant baculoviruses (*Methods S1*). In brief, Sf9 cells were seeded into 6-well plates, transfected with 0.8–2.0 µg of each construct using 4 µl CellFECTIN reagent (Gibco BRL) and 0.2 µg BaculoGold™ DNA (BD PharMingen) in serum-free media at 27°C for 4 h. TNM-FH + 10% FBS was then added and the cells were cultured at 27°C for 4–5 days to propagate baculovirus. Virus-containing medium was then harvested, centrifuged to remove cellular debris and stored at −80°C.

Following initial transfection, homologous recombinant baculoviruses for the various gene products were sequentially amplified to a high viral titer in Sf9 cells for subsequent large-scale protein expression. (*Methods S1*). In brief, for initial amplification, Sf9 cells were seeded into T75 flasks, recombinant baculovirus prepared as outlined above was added and the cells were propagated at 27°C for 4 days. The medium was harvested, centrifuged at 2,000×*g* to remove cellular debris and collected. The amplification step was then repeated.

Further amplification was achieved using suspension-cultured Sf9 cells. Cells were seeded into a spinner flask (BellCo), recombinant baculovirus was added to the flask and the cells were cultured at 27°C in a Cellgro Stirrer (Thermolyne) for 4 days. The cell debris was then removed by centrifugation and the supernatant containing baculovirus was stored at 4°C for up to 6 months.

### Expression of recombinant proteins in insect cells

The recombinant *ILK*-, *ILK^K220A^* ^-^, or α*-parvin*-encoding baculoviruses were infected into Sf9 insect cells. For production of the ILK/α-parvin complex, insect cells were co-transfected with equal amounts of *ILK* and *α-parvin* baculovirus. The recombinant proteins were expressed under the control of the PH promoter. Proteins were expressed in 5×10^8^ Sf9 cells (Invitrogen) in 500 ml of TNM-FH medium +10% FBS using an MOI of 5. Cells were grown in a spinner-flask with continuous stirring at 80 rpm and 27°C for 3 days. Cells were harvested by centrifugation at 1,000×*g* at 4°C for 5 min. The supernatant was carefully decanted and the cell pellets were processed immediately for affinity column purification.

### Purification of expressed proteins

Purification of the expressed ILK proteins (WT and K220A mutants) or the ILK/α-parvin complex was performed by affinity column chromatography. Recombinant proteins were isolated from Sf9 cells by mild sonication (3×30 s cycles) in 8× volume of Lysis Buffer (20 mM Tris-HCl, pH 7.5, 300 mM NaCl, 1 mM EDTA, 1% Triton-X-100, 1% NP40, 1× Protease Inhibitor Cocktail solution, 0.1 mM PMSF) and the lysate was cleared of cellular debris by centrifugation at 12,000 rpm for 10 min at 4°C. The cleared lysate was then applied to a glutathione-agarose column (Sigma-Aldrich). For large-scale purification, proteins were batch-bound to the beads by rotation at 4°C for 20 min. The beads were then collected by centrifugation at 1,000×*g* at 4°C for 3 min. The supernatant was carefully removed, the beads were resuspended in 15 ml of ice-cold High Salt Wash Buffer (50 mM Tris-HCl, pH 7.5, 500 mM NaCl, 1 mM EDTA, 0.1 mM PMSF) and loaded onto a column. All washes and elutions were performed at 4°C. The column was washed with 5 column volumes of High Salt Wash Buffer, followed by 5 column volumes of Low Salt Wash Buffer (50 mM Tris-HCl, pH 7.5, 50 mM NaCl, 1 mM EDTA, 0.1 mM PMSF). The bound GST-ILK was released from the column by elution with Glutathione Elution Buffer (50 mM Tris-HCl, pH 7.5, 50 mM NaCl, 1 mM EDTA, 10 mM glutathione, 0.05 mM PMSF). Fractions (0.5 ml) were collected, pooled and stored at −80°C. Purification of GST-ILK, GST-ILK^K220A^ and GST-ILK/His-α-parvin yielded approximately 50–60 µg of purified protein from 1 L of Sf9 insect cells. Stock solutions of GST-ILK, GST-ILK^K220A^ and GST-ILK/His-α-parvin, each at a concentration of 20 ng/µl, were prepared and stored at −80°C.

### Proteomics

Thirty micrograms of recombinant GST-ILK, including some GST alone, was resolved by 10% SDS-PAGE and stained with blue-silver Coomassie [60]. The stained lane was cut into three sections: between the GST band and the ILK band, the ILK band itself and above the ILK band. The region of the gel including the GST band and below was not analyzed since there are no known kinases smaller than GST (27 kDa). Proteins in the three gel slices were reduced, alkylated, digested with trypsin and analyzed by liquid chromatography-tandem mass spectrometry exactly as described [61]. The fragment spectra were searched against the SwissProt human protein database to confirm the presence of ILK. They were then searched against a library compiled from all Order *Lepidoptera* sequences in GenBank (37,435 sequences) using Mascot (v2.2). All identified *Lepidoptera* proteins meeting criteria for a 1% false discovery rate can be found in *Table S1*.

### Kinase activity assay

Kinase activity assays were performed using the following standard conditions: 30 ng of GST-ILK purified from Sf9 insect cells, 2 µg of the substrate GST-GSK3 crosstide (Cell Signaling Technology) and γ^32^P-labeled ATP (hot ATP:cold ATP  = 1∶1000; PerkinElmer) were added to the Kinase Reaction Buffer (25 mM Tris-HCl, pH 7.5, 5 mM β-glycerophosphate, 0.1 mM Na_3_VO_4_, 10 mM MgCl_2_) in a final volume of 20 µl. Reactions were carried out with constant shaking (120 rpm) for 30 min at 30°C in a TempMaster benchtop incubator/shaker (Fisher Scientific). Full-length LC_20_ (1.5 µg) was used as a substrate in certain experiments. In some experiments, various cations (MnCl_2_, CaCl_2_, ZnSO_4_) were substituted for MgCl_2_ in the reaction buffer. Their use and specific concentrations are indicated in the appropriate figures. Phosphorylated proteins were electrophoresed on 10–20% Tricine gradient gels (Invitrogen) using NuPAGE MES SDS running buffer (Invitrogen). Proteins were stained with Coomassie Blue and incorporation of radioactive phosphate was visualized using autoradiography. For quantification, electrophoresis was carried out in triplicate, individual bands were excised and samples were analyzed in a liquid scintillation counter. For experiments involving kinase inhibitors, stock inhibitors in DMSO were added directly to the kinase reaction at a concentration of 1 µM. Inhibitors included Dasatinib (Brystol-Myers Squibb), GF109203x (Sigma-Aldrich), MLCK inhibitor, CDK 1/5 inhibitor (Calbiochem), AMPK inhibitor compound C (Calbiochem) and QLT-2067 (QLT Inc).

### Western blot analysis

Western blotting was carried out as previously described [23], [24]. Primary antibodies used were rabbit-anti-α-parvin (Sigma), mouse-anti-ILK (BD Biosciences), rabbit-anti-phospho-Ser19-LC_20_ (Rockland Immunochemicals, Gilberville, PA) and rabbit-anti-phospho-Ser9-GSK3β (Cell Signaling Technology). All primary antibodies were used at a dilution of 1∶1000. Proteins were visualized by chemiluminescence using Supersignal (Pierce) or by fluorescence using the Odyssey system (Li-Cor Biosciences, Lincoln, NA). Adobe Photoshop was used for image adjustments. All image processing was applied to the whole image and levels were adjusted in a linear fashion.

### Michaelis-Menten kinetics

Substrate and enzyme velocity data were fit to a Michaelis-Menten model and V_max_ and K_m_ were calculated using the Michaelis-Menten model features in GraphPad Prism 5.00 for Windows (GraphPad Software, San Diego, CA). Substrate concentrations were varied and enzyme velocity (nmol substrate/µmol enzyme/min) was calculated from the original counts per minute (CPM) values and the specific activity of the radiolabeled ATP derived from liquid scintillation counting.

## Supporting Information

Methods S1 (0.04 MB DOC)

Figure S1 Mass spectrometry analysis of ILK. Fragment spectra were analyzed by liquid chromatography-tandem mass spectroscopy and searched against the SwissProt human protein data base to confirm the presence of ILK. Matching peptides are indicated in red. Sequence coverage exceeded 94%.
(3.68 MB TIF)

Figure S2 Effect of various kinase inhibitors on ILK kinase activity. Autoradiograph demonstrating ILK kinase activity in the presence of a variety kinase inhibitors. Reactions were carried out for 30 min using 30 ng of ILK and 10 mM MgCl2. LC20 was used as the substrate. Densitometric quantification of the bands is provided below the autoradiograph.
(3.46 MB TIF)

Table S1 (0.03 MB XLS)
