# Supplementary material for: Integrin-Linked Kinase Is a Functional Mn2+-Dependent Protein Kinase that Regulates Glycogen Synthase Kinase-3β (GSK-3β) Phosphorylation
Source: PLoS One. 2010 Aug 23;5(8):e12356. doi: 10.1371/journal.pone.0012356 (PMC2932980; doi:10.1371/journal.pone.0012356)
Supplement: Methods S1 — (0.04 MB DOC) [file pone.0012356.s001.doc]

**Supporting Materials and Methods**

*Cloning sites, primers and GST fusion protein sequence for ILK*

ILK-F182-EcoRI agaattcctatggacgacattttcactca

ILK-R1540-EcoRI agaattctacttgtcctgcatcttctcaag

GST-ILK protein sequence: total of 682 aa, including 226 aa of GST, 4aa of linker and 452 aa of wild-type ILK. The site **M**231 of the recombinant protein is equivalent to **M**1 in the native ILK sequence. The **K**220 is replaced by **A**220 in the mutant protein.

GST-ILK^WT^ (682 aa)

MSPILGYWKI KGLVQPTRLL LEYLEEKYEE HLYERDEGDK WRNKKFELGL

EFPNLPYYID GDVKLTQSMA IIRYIADKHN MLGGCPKERA EISMLEGAVL

DIRYGVSRIA YSKDFETLKV DFLSKLPEML KMFEDRLCHK TYLNGDHVTH

PDFMLYDALD VVLYMDPMCL DAFPKLVCFK KRIEAIPQID KYLKSSKYIA

WPLQGWQATF GGGDHPPKSD LVPRGSPGIP **M**DDIFTQCRE GNAVAVRLWL

DNTENDLNQG DDHGFSPLHW ACREGRSAVV EMLIMRGARI NVMNRGDDTP

LHLAASHGHR DIVQKLLQYK ADINAVNEHG NVPLHYACFW GQDQVAEDLV

ANGALVSICN KYGEMPVDKA KAPLRELLRE RAEKMGQNLN RIPYKDTFWK

GTTRTRPRNG TLNKHSGIDF KQLNFLTKLN ENHSGELWKG RWQGNDIVV**K**

VLKVRDWSTR KSRDFNEECP RLRIFSHPNV LPVLGACQSP PAPHPTLITH

WMPYGSLYNV LHEGTNFVVD QSQAVKFALD MARGMAFLHT LEPLIPRHAL

NSRSVMIDED MTARISMADV KFSFQCPGRM YAPAWVAPEA LQKKPEDTNR

RSADMWSFAV LLWELVTREV PFADLSNMEI GMKVALEGLR PTIPPGISPH

VCKLMKICMN EDPAKRPKFD MIVPILEKMQ DK

GST-ILK^K220A^ (681 aa)

MSPILGYWKI KGLVQPTRLL LEYLEEKYEE HLYERDEGDK WRNKKFELGL

EFPNLPYYID GDVKLTQSMA IIRYIADKHN MLGGCPKERA EISMLEGAVL

DIRYGVSRIA YSKDFETLKV DFLSKLPEML KMFEDRLCHK TYLNGDHVTH

PDFMLYDALD VVLYMDPMCL DAFPKLVCFK KRIEAIPQID KYLKSSKYIA

WPLQGWQATF GGGDHPPKSD LVPRGSPEF **M** DDIFTQCREG NAVAVRLWLD

NTENDLNQGD DHGFSPLHWA CREGRSAVVE MLIMRGARIN VMNRGDDTPL

HLAASHGHRD IVQKLLQYKA DINAVNEHGN VPLHYACFWG QDQVAEDLVA

NGALVSICNK YGEMPVDKAK APLRELLRER AEKMGQNLNR IPYKDTFWKG

TTRTRPRNGT LNKHSGIDFK QLNFLTKLNE NHSGELWKGR WQGNDIVV**A**V

LKVRDWSTRK SRDFNEECPR LRIFSHPNVL PVLGACQSPP APHPTLITHW

MPYGSLYNVL HEGTNFVVDQ SQAVKFALDM ARGMAFLHTL EPLIPRHALN

SRSVMIDEDM TARISMADVK FSFQCPGRMY APAWVAPEAL QKKPEDTNRR

SADMWSFAVL LWELVTREVP FADLSNMEIG MKVALEGLRP TIPPGISPHV

CKLMKICMNE DPAKRPKFDM IVPILEKMQD K

*Cloning sites, primers and His-tagged protein for α-parvin*

α-parvin-F50-BglII gagatctatggccacctccccgca

α-parvin-R1168-BglII gagatctcactccacgttacggt

N-terminal His-tag α-parvin (381 aa)

MHHHHHHGSM ATSPQKSPSV PKSPTPKSPP SRKKDDSFLG KLGGTLARRK

KAKEVSELQE EGMNAINLPL SPIPFELDPE DTMLEENEVR TMVDPNSRSD

PKLQELMKVL IDWINDVLVG ERIIVKDLAE DLYDGQVLQK LFEKLESEKL

NVAEVTQSEI AQKQKLQTVL EKINETLKLP PRSIKWNVDS VHAKSLVAIL

HLLVALSQYF RAPIRLPDHV SIQVVVVQKR EGILQSRQIQ EEITGNTEAL

SGRHERDAFD TLFDHAPDKL NVVKKTLITF VNKHLNKLNL EVTELETQFA

DGVYLVLLMG LLEGYFVPLH SFFLTPDSFE QKVLNVSFAF ELMQDGGLEK

PKPRPEDIVN CDLKSTLRVL YNLFTKYRNV E

*Transfection and amplification of recombinant baculovirus in Sf9 cells-* Purified pAcG2T-ILK, pAcSPG4T1-ILK^K220A^ and pAcSPHis-α-parvin plasmids were transfected separately with linear AcNPV DNA (BD PharMingen) into *Spodoptera frugiperda* (Sf9) cells (Invitrogen) to produce recombinant baculoviruses. Specifically, Sf9 cells were seeded into 6-well plates at 8 x 10^5^ cells per well of TNM-FH insect cell medium (JRH Biosciences) containing 10% FBS (TNM-FH + 10% FBS) and were allowed to attach at 27 °C for 30 min. For each transfection, the following mixture was prepared and incubated at room temperature for 15 min: 0.2 μg BaculoGold^TM^ DNA (BD PharMingen), 0.8 - 2.0 αg baculovirus transfer vector DNA, 4 μl CellFECTIN reagent (Gibco BRL) and 0.6 ml serum-free TNM-FH medium (JRH Biosciences). Cell layers were washed, lipid-DNA transfection complexes were added and cells were shaken at 60 rpm at 27 °C for 4 h. TNM-FH + 10% FBS was then added and the cells were cultured at 27 °C for 4 - 5 days to propagate baculovirus. Virus-containing medium was then harvested and centrifuged at 14,000 rpm for 5 min to remove debris. The supernatant containing recombinant baculovirus was collected and stored at -80 °C.

For large scale amplification of recombinant baculoviruses, Sf9 cells were seeded into T75 flasks at 6 x 10^6^ cells/flask, allowed to attach for 15 min and refed with fresh TNM-FH medium + 10% FBS. 300 μl of low titer recombinant baculovirus, prepared as outlined above, were added and the cells were incubated at 27 °C for 4 days. The medium was harvested, centrifuged at 2,000x g in a Beckman Avanti GS-6R centrifuge with rotor GH3.8 at room temperature for 5 min to remove cellular debris and the supernatant (recombinant baculovirus) was collected. The amplification step was then repeated.

Further amplification was achieved using suspension-cultured Sf9 cells. 300 ml of suspension-cultured Sf9 cells were prepared by seeding 1 x 10^6^ cells/ml into a spinner flask (BellCo). 3 ml of amplified recombinant baculovirus was then added to the flask and the cells were cultured at 27 °C in a Cellgro Stirrer (Thermolyne) with a stirring rate of 70 - 80 rpm for 3 - 4 days. The cell debris was removed by centrifugation at 2,000x g in a Beckman Avanti J-25 using rotor JLA10500 at 4 °C for 10 min. The supernatant containing baculovirus was collected and the virus was stored 4 °C for up to 6 months.
