# Supplementary figures and images for: Integrin-Linked Kinase Is a Functional Mn2+-Dependent Protein Kinase that Regulates Glycogen Synthase Kinase-3β (GSK-3β) Phosphorylation
Source: PLoS One. 2010 Aug 23;5(8):e12356. doi: 10.1371/journal.pone.0012356 (PMC2932980; doi:10.1371/journal.pone.0012356)

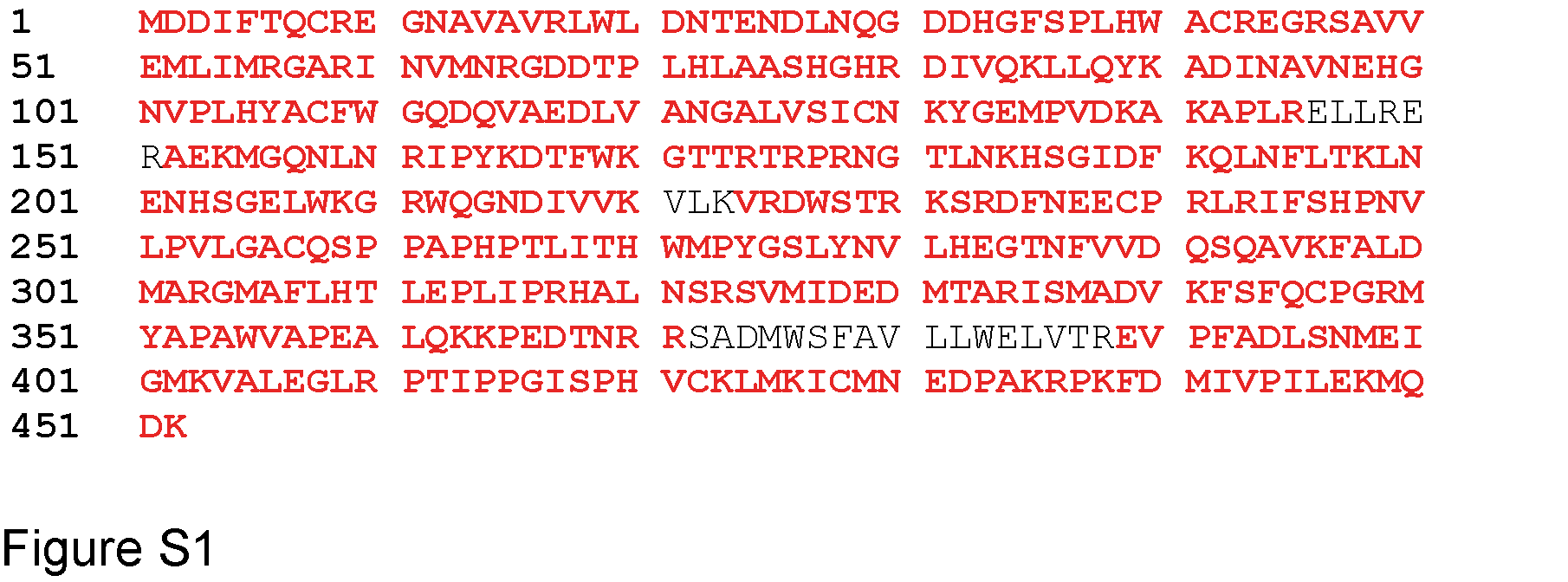

Supplement: Figure S1 — Mass spectrometry analysis of ILK. Fragment spectra were analyzed by liquid chromatography-tandem mass spectroscopy and searched against the SwissProt human protein data base to confirm the presence of ILK. Matching peptides are indicated in red. Sequence coverage exceeded 94%. (3.68 MB TIF) [file pone.0012356.s002.tif]

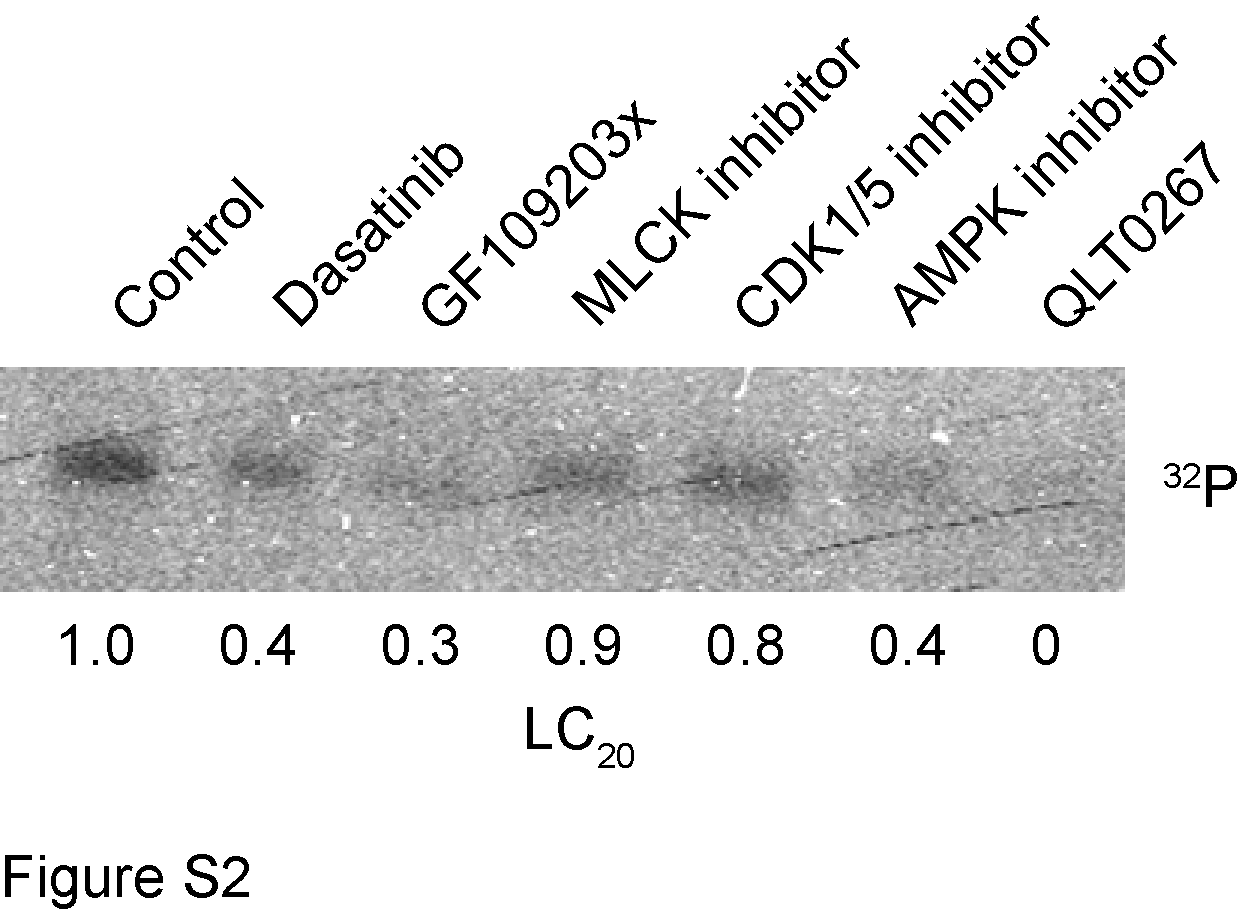

Supplement: Figure S2 — Effect of various kinase inhibitors on ILK kinase activity. Autoradiograph demonstrating ILK kinase activity in the presence of a variety kinase inhibitors. Reactions were carried out for 30 min using 30 ng of ILK and 10 mM MgCl2. LC20 was used as the substrate. Densitometric quantification of the bands is provided below the autoradiograph. (3.46 MB TIF) [file pone.0012356.s003.tif]
